# Negative pressure pulmonary edema: a case report and literature review

**DOI:** 10.3389/fmed.2024.1467541

**Published:** 2024-12-04

**Authors:** Jian Wu, Hua Yuan, Zhiqiang Guo, Qiupeng Feng, Jin Ma

**Affiliations:** Department of Emergency Medicine, Affiliated Kunshan Hospital of Jiangsu University, Kunshan, China

**Keywords:** negative pressure pulmonary edema, non-cardiogenic pulmonary edema, post-anesthetic complication, patient prognosis, case report

## Abstract

Negative pressure pulmonary edema (NPPE) is a form of non-cardiogenic pulmonary edema triggered by a swift increase in negative intrapleural pressure due to upper airway obstruction and represents a potential cause of acute respiratory failure. This study documents a case of NPPE post-endotracheal extubation subsequent to general anesthesia. The patient, a young female, underwent a “laparoscopy-assisted unilateral salpingectomy” under general anesthesia for an ectopic pregnancy. Immediately post-extubation, the patient exhibited a sudden decline in oxygen saturation and tachypnea. Pink frothy secretions were suctioned from the oral and nasal cavities. Swift interventions, including oxygen therapy, non-invasive ventilation, diuretics, and corticosteroids, were administered. The patient’s condition was effectively managed, and after 6 days of treatment, she was discharged from the hospital following full recovery.

## Introduction

Negative pressure pulmonary edema (NPPE) represents non-cardiogenic pulmonary edema precipitated by a rapid escalation in intrathoracic negative pressure due to acute or chronic upper airway obstruction, potentially resulting in life-threatening hypoxemia (1). Since the initial documentation of NPPE (2), its incidence and mortality rates have demonstrated variability across various reports (1, 3). NPPE arises from multiple factors, with laryngospasm post-anesthetic extubation being the predominant cause. The fundamental pathophysiological mechanism entails the production of significant inspiratory pressure by patients to counteract upper airway obstruction, thereby causing a progressive rise in intrathoracic negative pressure and subsequent increase in pulmonary capillary pressure (1, 4). Clinically, NPPE manifests as severe respiratory distress, hypoxia, and expectoration of pink frothy sputum, with imaging typically indicating cardiogenic pulmonary edema (1, 5). Standard treatments encompass airway management, oxygen supplementation, positive pressure ventilation, and intensive care unit (ICU) care (3). Additionally, many patients achieve recovery through supportive care alone (1), although the use of pharmacological interventions remains a subject of debate (6). This report delineates a case of NPPE following general anesthesia and tracheal extubation, which was successfully managed in the Emergency Department of Kunshan Hospital Affiliated to Jiangsu University.

## Case report

**Figure 1:**
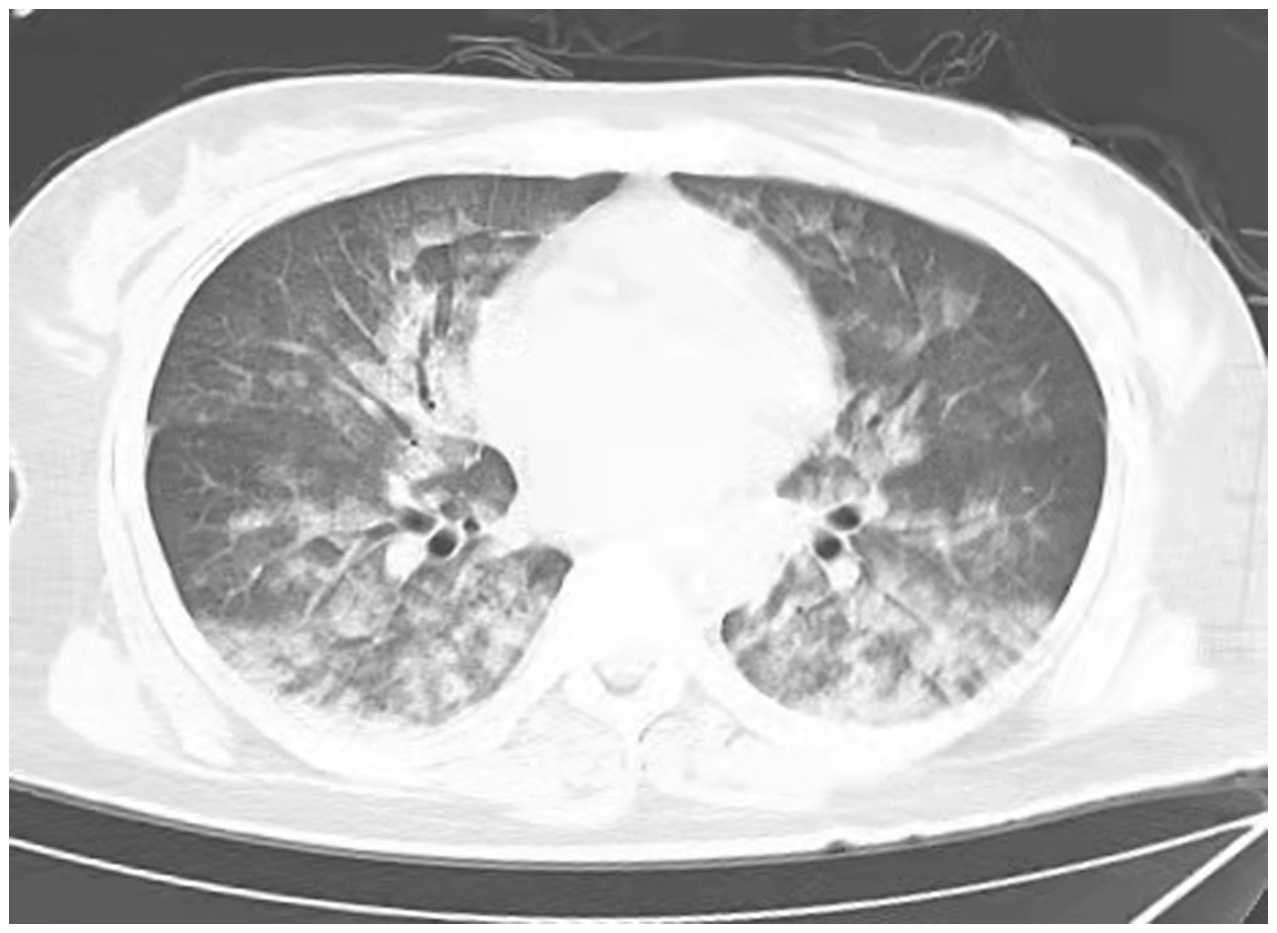
Chest CT scan depicting increase in diffuse density of the lungs, bilateral ground glass opacities, and dorsal consolidation of lungs because of pulmonary infiltrates, supporting acute pulmonary edema at 0.15 h after extubations. (Extensive, patchy ground-glass opacities and cloud-like shadows were observed in both lungs).

**Figure 2:**
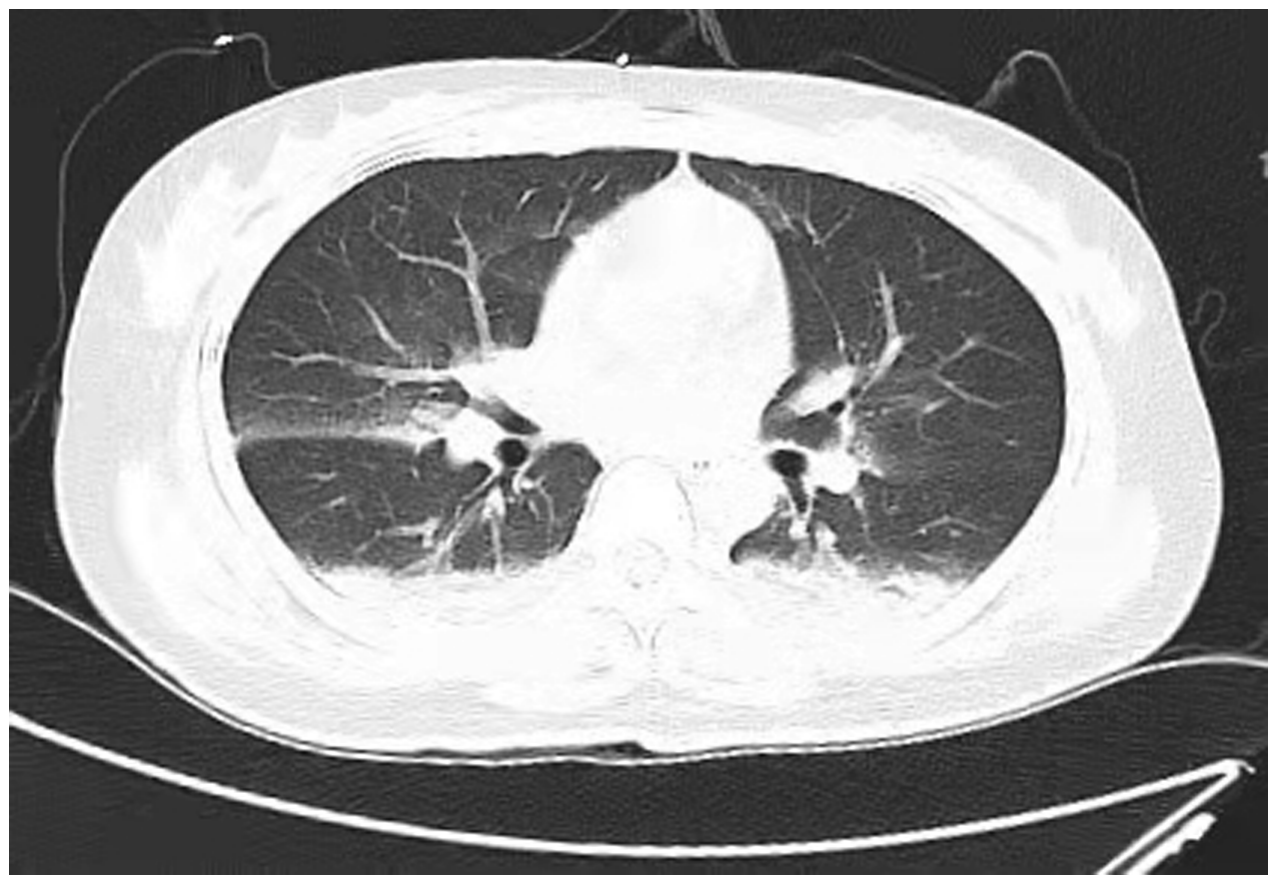
Chest CT scan depicting most of the lung infiltrates resolved at 6 h after extubations. (Scattered cloud-like opacities with blurred margins are observed in both lungs).

**Figure 3:**
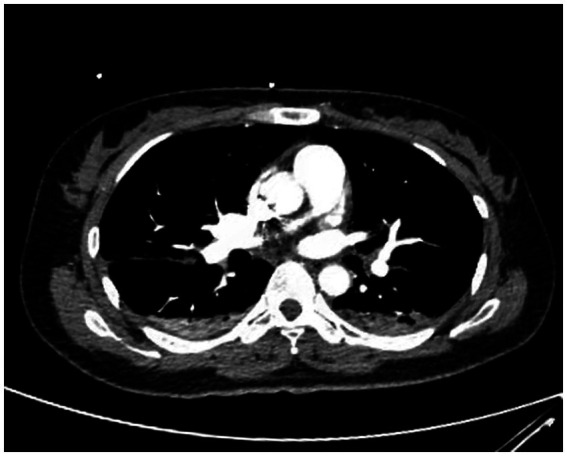
Contrast-enhanced CT showed no evidence of pulmonary embolism. (Pulmonary embolism angiography reveals two areas of pneumonia-related changes and extensive alveolar pulmonary edema).

**Figure 4:**
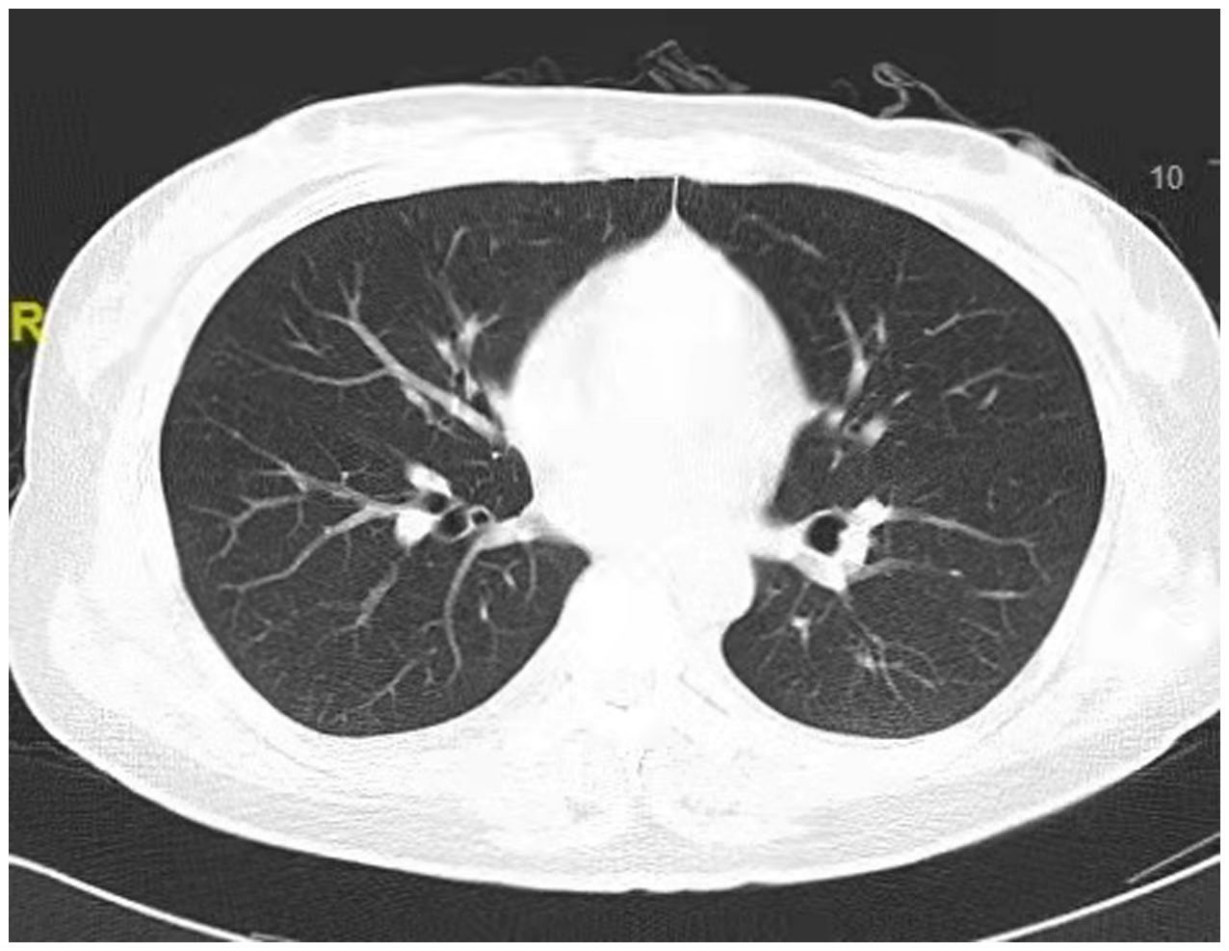
Chest CT scan depicting clear lung fields, indicating normal performance of the lungs at 6 days after extubations.

A 38-year-old married female with no previous history of cardiac disease, asthma, pneumonia, or allergies to food or medication presented with a ruptured ectopic pregnancy. On March 9, 2022, at 19:45, she underwent an emergency laparoscopic-assisted salpingectomy under general anesthesia at a local hospital. All preoperative evaluations, including laboratory tests, imaging studies, and physical examinations, were within normal limits. The surgery concluded at 21:00 without complications, and the anesthesia was satisfactory. Intraoperative fluid replacement included 1,000 mL of normal saline. At 21:10, the endotracheal tube was removed, and the patient was alert with no complaints. At 21:15, cardiac monitoring indicated a decrease in peripheral oxygen saturation (SpO_2_) to approximately 75%. The patient reported chest tightness and dyspnea, with pink frothy sputum. Physical examination revealed clear consciousness, tachypnea, and bilateral pulmonary rales. Immediate oxygen therapy via nasal cannula was started. Arterial blood gas analysis revealed: pH 7.31, partial pressure of arterial oxygen (PaO_2_) 83 mmHg, partial pressure of arterial carbon dioxide (PaCO_2_) 47 mmHg. A 12-lead electrocardiogram displayed sinus rhythm without signs of acute ischemia or infarction. After administration of 40 mg methylprednisolone and 20 mg furosemide intravenously, oxygen therapy was switched to a face mask, resulting in a SpO_2_ of approximately 95%. Due to ongoing chest tightness and dyspnea, the patient was transferred to our emergency department. Upon admission, the patient reported chest tightness and mild dyspnea. Vital signs were: temperature 37.3°C, pulse 100/min, respiratory rate 26/min, blood pressure 108/72 mmHg, SpO_2_ 98% (with face mask oxygen). Physical examination revealed bilateral pulmonary rales, with normal cardiac and abdominal findings and no lower extremity edema. Non-invasive positive pressure ventilation was promptly initiated, along with 20 mg furosemide intravenously. Arterial blood gas analysis showed: pH 7.34, PaO_2_ 127 mmHg, PaCO_2_ 47 mmHg, white blood cell count 16.47*109/l, neutrophil percentage 96.6%, hemoglobin 107 g/L, platelet count 202*109/l, D-Dimmer 1.98 mg/L fibrinogen equivalent units, N-terminal pro-B-type natriuretic peptide 86.5 pg/mL. Chest computed tomography (CT) revealed bilateral pulmonary exudative changes consistent with pulmonary edema (Figure 1). After 6 h, the patient’s symptoms improved, with no dyspnea or chest pain and only a mild cough without sputum. Repeat arterial blood gas analysis showed: pH 7.46, PaO_2_ 285 mmHg, PaCO_2_ 45.9 mmHg. A follow-up chest CT at 12 h demonstrated significant resolution of the bilateral pulmonary exudative changes (Figure 2). Contrast-enhanced CT showed no evidence of pulmonary embolism (Figure 3). The patient’s respiratory status improved over the following days as the pulmonary edema resolved. She was discharged after 6 days without any complaints, and a final chest CT showed no significant abnormalities (Figure 4). A one-month follow-up revealed no residual symptoms.

## Discussion

First documented in 1973, NPPE has since been reported numerous times with varying incidence rates (2). The reported incidence of NPPE ranges from 0.1 to 12% (7–10), although the true incidence remains uncertain. Given the frequent occurrence of upper airway obstruction during the perioperative period, it is hypothesized that the actual incidence is likely much higher, with many cases potentially misdiagnosed or undiagnosed. Mortality rates also exhibit variability. Initially, the mortality rate for NPPE was reported to range from 11 to 40%. Subsequent literature reviews have indicated a mortality rate of 2% (11, 12). A recent systematic review of NPPE in adult otolaryngologic surgeries reported a mortality rate of 5%, identifying age and ICU admission as primary risk factors for mortality (3).

Airway obstruction from various factors is directly responsible for NPPE, with laryngospasm most frequently occurring during recovery from general anesthesia. In adults, 55% of NPPE cases are caused by perioperative laryngospasm, and studies indicate that the incidence of laryngospasm during extubation is 0.87% (1). Additionally, oropharyngeal surgery, obstructive respiratory distress, obesity, and laryngeal mask displacement significantly contribute to NPPE. Research involving 86,561 patients (13) revealed that active smokers and those undergoing endotracheal intubation for general anesthesia face a significantly higher risk of developing NPPE post-extubation in the operating room. NPPE has also been reported following extubation after general anesthesia for surgeries beyond the oropharyngeal region. This case involves a patient who underwent a laparoscopic-assisted unilateral salpingectomy under general anesthesia. We believe that the cause of NPPE in this patient was likely laryngospasm following general anesthesia. Laryngospasm is the most common cause of NPPE, accounting for 55% of cases. In this instance, the patient was a young, previously healthy woman with no history of lung disease who experienced oxygen desaturation and dyspnea after extubation post-surgery, further supporting laryngospasm as the likely trigger.

The pathogenesis of NPPE is primarily attributed to the generation of substantial negative intrapleural pressure. In adults, inspiratory efforts against a closed upper airway can generate negative pressures up to approximately −140 mmHg (4). This degree of negative pressure is sufficient to significantly enhance venous return to the right heart and cause the interventricular septum to shift towards the left ventricle, thereby decreasing stroke volume. Concurrently, as venous return increases, more blood enters the pulmonary circulation, resulting in elevated hydrostatic pressure within the pulmonary circulation. The pulmonary microvascular system promotes fluid extravasation from the vascular bed into the interstitium (14). The formation and resolution of NPPE pulmonary edema are linked to the low protein concentration in the pulmonary edema fluid of most NPPE patients, suggesting that elevated hydrostatic pressure is responsible for NPPE development. Although negative intrathoracic pressure is the primary factor in NPPE pathogenesis, other factors are also significant. Ventilatory efforts against an obstructed airway ultimately lead to hypoxia and acidosis, which elevate pulmonary vascular resistance and adversely affect alveolar-capillary integrity. Moreover, significant inspiratory efforts trigger a high adrenergic response, further increasing pulmonary vascular resistance and directly promoting blood redistribution from the systemic to the pulmonary circulation (15).

Respiratory distress, hypoxia, cyanosis, pink frothy sputum, and hemoptysis are typical symptoms and signs of NPPE. Key diagnostic criteria include: (1) a history of the upper airway obstruction; (2) sudden onset of respiratory difficulty, hypoxia, and hypercapnia within minutes to hours after obstruction relief; (3) presence of pink frothy sputum; (4) radiological findings: X-ray showing diffusely increased density, widened vascular shadows, and bilateral, central alveolar and interstitial infiltrates. Given its similarity to aspiration pneumonia during anesthesia and other causes of pulmonary edema, clinicians must be vigilant in differential diagnosis. When the diagnosis is uncertain, it is crucial to exclude causes such as cardiogenic pulmonary edema, minor reflux aspiration, acute respiratory distress syndrome, allergic pulmonary edema, and neurogenic pulmonary edema. In this case, the patient presented with a clinical manifestation of coughing up frothy pink sputum, which can easily lead clinicians to misdiagnose NPPE as left heart failure, cardiogenic pulmonary edema, or aspiration pneumonia in an emergency, thereby delaying optimal treatment. In particular, imaging results are useful in differentiating NPPE from cardiogenic pulmonary edema (5). NPPE typically presents with prominent bilateral perihilar alveolar infiltrates, whereas cardiogenic pulmonary edema follows a more interstitial pattern and usually shows evident blood flow diversion to the lung apices (1). With the rapid advancement of bedside ultrasound technology, Zhang et al. (16) reported a case of rapid diagnosis and treatment of NPPE in a 35-year-old female patient using bedside ultrasound. This patient had a history of general anesthesia for a ruptured ectopic pregnancy. Considering symptoms such as rapid breathing and decreased oxygen saturation and differentiating from pulmonary embolism, enhanced CT showed no significant signs of pulmonary embolism. Given the patient’s profile as a young female without underlying cardiac disease, the condition’s changes occurring 5 min after extubation, and relevant auxiliary examination results, acute NPPE was diagnosed. Furthermore, acute NPPE can manifest as quickly as within seconds or as late as 4 h after the relief of airway obstruction.

The management of NPPE is primarily aimed at symptomatic relief, focusing on enhancing oxygenation and reducing pulmonary edema. The severity, progression, and outcome of NPPE are determined by the duration of obstruction. The decision to reintubate is crucially dependent on the ability to maintain effective oxygenation. Treatment involves vigilant monitoring, ensuring airway patency, oxygen therapy, and respiratory support through endotracheal intubation or non-invasive ventilation (1, 17, 18). In the presented case, the patient did not exhibit significant airway obstruction upon admission, and the condition had markedly improved with non-invasive ventilation prior to hospitalization, thereby avoiding endotracheal intubation. The underlying pathology causing pulmonary edema must be considered when contemplating intubation. When mechanical ventilation is necessary, lung-protective ventilation strategies are advised, even for patients without acute respiratory distress syndrome (1). While diuretics are standard for cardiogenic pulmonary edema, they play a secondary role in NPPE management. Some studies suggest that the use of furosemide should be etiology-dependent (1, 4), whereas others argue that its use is controversial due to the risks of hypoperfusion and hypovolemia. However, in this instance, the patient’s symptoms significantly improved following furosemide administration. Additionally, most NPPE cases can be effectively managed with ventilatory support, such as continuous positive airway pressure, bilevel positive airway pressure, or mechanical ventilation. In severe cases, venovenous extracorporeal membrane oxygenation has been successfully employed (19).

In conclusion, NPPE is recognized as a life-threatening post-anesthetic complication, necessitating prevention and early recognition as essential components of management. Early diagnosis and timely treatment result in improved outcomes. Perioperative patients should be vigilantly monitored for NPPE, particularly in the absence of a prior cardiac history or cardiovascular risk factors. In instances where acute pulmonary edema develops during recovery from general anesthesia, NPPE should be highly suspected. Additionally, immediate and assertive intervention is critical for enhancing patient prognosis.

## Data Availability

The raw data supporting the conclusions of this article will be made available by the authors, without undue reservation.
